# Progress in Surgery

**Published:** 1902-11-22

**Authors:** 


### Progress in Surgery.

### OTOLOGY.

**Intra-nasal Treatment in Non-suppurative Middle-ear Disease.**—P. McBride [1] introduced a discussion on this subject at the Manchester meeting of the British Medical Association. The following are some of the views he expressed: Wherever adenoids are present with catarrhal deafness they should be removed, for a small amount of adenoid tissue may press upon the Eustachian tubes and seriously affect hearing. The adenoids may cause no other symptoms whatever. On the other hand, more bulky adenoids situated more centrally may not affect the ears. After the operation hearing may be improved, although the tympanic membrane still remains in-drawn. In sclerosis of the middle ear, which may be met with in the pure form in children, if adenoids are present, their removal is not likely to prove of benefit. If the aural affection is a mixed one of catarrh and sclerosis then possibly some benefit will ensue from the removal of adenoids. The writer takes a sceptical attitude with regard to the benefits to hearing likely to accrue from operations on the nose. In aural sclerosis without catarrh he is practically certain that no improvement will follow such operations. Further, the writer states that he does not remember cases in which the removal of the posterior ends of the turbinate bones has been shown to do more for the ears than could be accomplished by inflation. On general grounds marked bilateral nasal obstruction should be dealt with by operation, but if the obstruction is unilateral the chief indication for operation will be the amount of nasal discomfort experienced by the patient, whether the patient be deaf or not. If nasal obstruction, associated with a diffuse congestion or a general catarrhal tendency of the upper respiratory tract, co-exists with catarrhal middle-ear disease, then operation would be justified. Dundas Grant thought it was a good practical rule that operations should not be performed on the nose on account of chronic non-suppurative disease of the middle ear, unless they were called for on account of the nasal obstruction. Lambert Lack advanced the dictum that nasal obstruction *per se* was never a cause of deafness. In cases of deafness with indrawn drums, associated with nasal obstruction, a certain amount of Eustachian obstruction would invariably be present. Surely Eustachian catarrh and obstruction, with or without more or less middle-ear catarrh, was the cause of the ear trouble in these cases. Children with simple nasal stenosis were usually not deaf. Children and adults with adenoids were often deaf, but only when the tubes were blocked.

**Chronic Non-suppurative Ear Diseases.**—Donald M. Bristow [2] has related a case which well illustrates the value of perseverance in the treatment of the above affection. The patient, a man of 22, who had been unable to hear with his right ear since an illness, which he was told was typhus fever, 14 years previously. The membrana tympani was retracted and pale but not thickened, and the short process of the malleus was very prominent. A 36-inch watch could not be heard on pressure. The tuning-fork reactions were those of middle-ear deafness and the Eustachian tube was patent. Energetic inflation through the catheter gave no result. Next day acoustic massage was performed, then Lucas's pressure probe used, and finally the ear was inflated with the catheter. After this the watch could be heard faintly on contact. For three weeks the patient was treated every second day by the pressure probe followed by catheterisation. On the twenty-fourth day he could hear the watch at 36 inches, and every word of a conversation 20 feet away. The author considers that the deafness was due to simple fibrous anchylosis between the ossicles, and that the bands were stretched or broken by means of the pressure probe. Macleod Yearsley [3] has contributed a paper on the treatment of non-suppurative ear disease by the application of super-heated air. The writer refers to a previous paper by G. W. Hopkins (Ohio) on the subject. The thought occurred to Hopkins to try this treatment in cases of anchylosis of the ossicles. He has now treated 62 cases and only had four failures, all of which were in very old people with extrusive labyrinthine involvement. Yearsley has tried the method also with favourable results, an example of which is detailed. The apparatus used by Hopkins consists of a simple room-heater operating either by gas or oil, and having a funnel-shaped top which sends hot air through a canvas sleeve to the ear. The ear is packed and covered by gauze, which takes up all moisture as rapidly as it is formed and prevents burning and makes the application of very hot air easy and free from discomfort. The case recorded by Yearsley was that of a police sergeant, aged 36, who for two years had had deafness in both ears, with buzzing tinnitus and paracusis Willisii. The voice was only heard when raised, and the watch not at all, on the left side, and only on contact on the right. For more than two months he was treated by means of the catheter, the chloride of ammonium inhaler, injections of parolein, and every other method that could be suggested. The right ear was slightly improved. Then hot air was tried of a temperature varying between 370° and 420°. In the later applications the packing of the ear was discarded. After each application the catheter was employed, and the hand masseur. Up to the fifteenth application the improvement was very gradual, then it became more rapid. At the time of writing the patient could hear a whisper at 86 inches. The author is treating a number of other cases on the same lines, and all of them seem to be improving. A. Bronner, at the Manchester Association meeting, showed an apparatus for the intra-tympanic hot-air treatment of certain forms of catarrh of the middle ear. It consisted of a metal air-tight cylinder, lined with asbestos, which fitted over a Prometheus heating-oven. The heat was generated by the electric current passing through specially prepared thin mica sheets, and the heat could be regulated. The cylinder was connected with a large hand-pump, or with an American air receiver, and thus a constant stream of air could be produced. The hot air was blown through an ordinary Eustachian catheter into the middle ear for from a half minute to five minutes, according to the nature of the case. In some chronic cases of tinnitus the results were excellent. Chalmers Watson [4] has contributed another paper, dealing with the treatment of ear disease with myelocene, a special preparation from bone marrow. We abstracted Watson's original paper in these columns. There it was shown that out of 15 cases 60 per cent. had received practical benefit. The experience with subsequent cases has confirmed the favourable opinions then formed as to the value of this mode of treatment. It is alleged that by it "we can improve the hearing in many cases of chronic non-suppurative middle ear disease to an extent considerably above that attainable by present methods." The coexistence of internal ear disease does not contraindicate the treatment. Thus, in two cases, the tuning fork was not heard on the mastoid before treatment, but after treatment the same tuning fork became clearly audible. The author remarks upon the unreliability of certain voice tests in the recognition of changes in hearing capacity. An example is given in which hearing for the conversational voice had much deteriorated in a period of three months, and yet the whisper test showed actually a slight improvement. The principles of treatment in this affection are as follows, briefly stated, viz. :—1. The promotion and maintenance of a more vigorous circulation in the middle ear. 2. The maintenance of thorough aëration of the naso-pharynx and middle ear. 3. The restoration of a greater degree of flexibility to the tympanic membrane and the associated structures. For the latter purpose it was reasonable to expect that an oil which is an internal animal secretion would possess more value than other oils. The mode in which myelocene acts had not yet been satisfactorily determined. Possibly it acted partly of increasing vascular activity and partly in virtue by an enzyme or other active principle found in the bone marrow. H. O. Reik [5] has written on catarrhal otitis (non-suppurative) as a factor in the etiology of facial paralysis. The points the author particularly lays stress upon are the following :—1. If exposure to cold is the cause of most cases of facial paralysis it probably most commonly acts by producing otitis media, and the nerve is then involved either by direct extension of the inflammatory process or by pressure of the exudate. 2. If this is admitted, paracentesis of the tympanic membrane is the most rational treatment, for by it the tympanic cavity may be emptied of its abnormal contents. 3. In any case of facial paralysis the ear should be examined.

**Perforation of the Membrana Tympani from a Lightning Stroke.**—H. Macnaughton Jones [6] has recorded a case of this kind. The following are the chief points in the history : An officer was sitting on the floor of his tent drinking tea from an iron cup. A storm was passing at the time. He then lost consciousness for a time, and on recovering consciousness, perhaps after two hours, he found himself paralysed, as he thought, with the exception of his left arm. The hair on his left temple was burned and that on the pubes. There was an abrasion of the right breast, a stripe running from this down to the right thigh, and burns on the shoulders. The hearing of the left ear was seriously affected. When seen by Jones the entire tympanic membrane was injected and of a deep red colour. There were two perforations. The most troublesome symptom was a rather loud tinnitus. A weak solution of nitrate of silver was periodically applied to the membrane, the ear protected with antiseptic gauze, and Lucas's inflator occasionally used. Three months later hearing was greatly improved. The writer states that the case is the only one of which he is aware in otological literature in which perforation of the membrana tympani has been found as the result of lightning.

[1] Brit. Med. Jour, Aug. 30, 1902, p. 602. [2] Vide Abst. Jour. L.R.&O , June 1902, p. 332. [3] Vide Reprint Monthly Homœopath. Rev., May 1, 1902, p. 284. [4] Brit. Med. Jour., Aug. 30, 1902, p. 612. [5] Vide Abst. Jour. L.R.&O., June 1902, p. 334. [6] Jour. L.R.&O., July 1902, p. 344.

